# New By-Law of the Pan-American Medical Congress

**Published:** 1893-04

**Authors:** 


### NOTICES.

### New By-Laws of the Pan-American Medical Congress.

LANGUAGES: *By-Law IX.* Papers may be read in any language, providing that authors of the same shall furnish the Secretary General with an abstract not exceeding six hundred words in length, in either of the official languages (English, Spanish, French or Portuguese), by not later than July 10th, 1893, and providing, further, that a copy of each such paper shall be furnished in either of the official languages, at or before the time of the meeting to the Secretary of the Section before which the same shall be read. Remarks upon papers may be made in any language, providing that members making such remarks shall furnish a copy of the same in either of the official languages, before the adjournment of the session.

PUBLICATION: *By-Law X.* All papers read, either in full or by title, shall be immediately submitted for publication in the transactions, (Special Regulation 3), but authors may retain copies and publish the same at their pleasure after the adjournment of the Congress.

CONSTITUENT ORGANIZATIONS: *By-Law XI.* All Medical, Dental and Pharmaceutical organizations, the titles of which have been transmitted with approval to the Committee on Organization or which may hereafter be transmitted with approval to the Executive Committee by any member of the International Executive Committee, each for his own country, shall be subject to election by the Executive Committee, approved by the President, as constituent bodies of the First Pan-American Medical Congress, and each organization thus constituted shall have the right to designate as delegates all of its members attending the Congress, but no such organization shall meet at the time and place of meeting of the Congress as a distinct body, providing, that the Secretary of each such constituent body shall furnish a list of officers and a statement of the number of members of his respective organization to the Secretary-General not later than sixty (60) days before the meeting of the Congress, and shall forward a list of delegates chosen, to reach the Secretary-General before the opening of the Congress.

By the Executive Committee.

February 22, 1893.

